# A canine model of tracheal stenosis induced by cuffed endotracheal intubation

**DOI:** 10.1038/srep45357

**Published:** 2017-03-28

**Authors:** Zhuquan Su, Shiyue Li, Ziqing Zhou, Xiaobo Chen, Yingying Gu, Yu Chen, Changhao Zhong, Minglu Zhong, Nanshan Zhong

**Affiliations:** 1 Sate Key Laboratory of Respiratory Disease, Guangzhou Institute of Respiratory Disease, The First Affiliated Hospital of Guangzhou Medical University, Guangzhou, China; 2 Pathology Department, The First Affiliated Hospital of Guangzhou Medical University, Guangzhou, China

## Abstract

Postintubation tracheal stenosis is a complication of endotracheal intubation. The pathological mechanism and risk factors for endotracheal intubation-induced tracheal stenosis remain not fully understood. We aimed to establish an animal model and to investigate risk factors for postintubation tracheal stenosis. Beagles were intubated with 4 sized tubes (internal diameter 6.5 to 8.0 mm) and cuff pressures of 100 to 200 mmHg for 24 hr. The status of tracheal wall was evaluated by bronchoscopic and histological examinations. The model was successfully established by cuffed endotracheal intubation using an 8.0 mm tube and an intra-cuff pressure of 200 mmHg for 24 hr. When the intra-cuff pressures were kept constant, a larger sized tube would induce a larger tracheal wall pressure and more severe injury to the tracheal wall. The degree of tracheal stenosis ranged from 78% to 91% at 2 weeks postextubation. Histological examination demonstrated submucosal infiltration of inflammatory cells, hyperplasia of granulation tissue and collapse of tracheal cartilage. In summary, a novel animal model of tracheal stenosis was established by cuffed endotracheal intubation, whose histopathological feathers are similar to those of clinical cases of postintubation tracheal stenosis. Excessive cuff pressure and over-sized tube are the risk factors for postintubation tracheal stenosis.

Endotracheal intubation is an emergency procedure frequently performed in critically injured, ill, or anesthetized patients to facilitate mechanical ventilation. However, prolonged tracheal intubation and excessive cuff pressure may potentially induce postintubation tracheal stenosis, which can cause breathing difficulties, suffocation, and even life-threatening. At present, cuffed tracheal tubes are used for all adults and older children^1^. Cuff is an inflatable balloon at one end of endotracheal tube with the function to prevent leakage around the trachea during ventilation. The cuff pressure should be high enough to avoid aspiration and leakage but not to interrupt the blood flow in the trachea^2^. Accumulating evidences have showed that endotracheal tube cuff pressure plays a crucial role in the development of postintubation tracheal stenosis^3^. Compared with conventional high-pressure low-volume cuff, current high-volume low pressure (HVLP) cuffs have a larger contact area between cuff and trachea, consequently causing a smaller tracheal wall pressure and reducing the compression injury on tracheal wall. Even though wide use of HVLP cuffs has reduced the incidence of intubation-induced tracheal injury^4^, however, intubation-induced tracheal injury still can not completely prevented. Previous study reported that HVLP cuffs are unable to be maintained at a stable low pressure during the entire period of intubation, such that in 73% of patients the cuff pressure becomes higher than the original pressure^5^. The incidence of ischemic tracheal lesions caused by HVLP cuffs varies from 31–95% of critical care patients, and 1–4% patients develop various degrees of tracheal stenosis after extubation^6^,^7^,^8^.

With recent advancement of interventional pulmonology, a variety of treatments such as balloon dilatation, thermal ablation, cryotherapy and stent implantation have significantly improve the therapeutic efficacy of tracheal stenosis^9^. Nevertheless, restenosis remains unavoidable in some patients, usually resulting in a poor prognosis^10^. Therefore, developing new therapeutic strategy is required. At present, the pathological mechanism and risk factors for endotracheal intubation-induced postintubation tracheal stenosis are still not fully understood. To further elucidate the pathogenesis mechanism and develop effective prevention and treatment methods, it is necessary to establish an appropriate animal model of tracheal stenosis similar to clinical condition.

Various animal models of tracheal stenosis have already been established through different methods^11^,^12^,^13^,^14^, including thermal ablation, trachea incision, and chemical damage. However, these methods are complicated and have a high mortality rate, and thus have been found limited use in basic research. More importantly, these models are not consistent with the most common clinical cause of tracheal stenosis, postintubation tracheal stenosis. A tracheal stenosis animal model established through tracheal intubation will more closely resemble that seen clinically, and can facilitate further exploration of pathogenesis and treatment. Since 1980 s, canine^15^ and ferret^16^ models of tracheal stenosis induced by prolong intubation have been reported. However, these models are limited by a time-consuming, lack of stability, and low reproducibility. Recently, Kumar *et al*.^17^ and Lee *et al*.^18^ have reported rabbit models of tracheal stenosis induced by tracheal intubation. However, all these models are established by uncuffed tracheal tubes which are used only for children clinically^1^. In addition, since excessive cuff compression on the tracheal wall is the main cause of ischemic injury and tracheal stenosis, these models cannot simulate the most common type of tracheal stenosis. So far, there is still no study on establishment of animal model of tracheal stenosis using cuffed endotracheal tube.

Beagles are appropriate for animal models of trachea-related diseases because the anatomical structure, inner diameter and physiological characteristics of the trachea are similar between beagles and adult humans^12^,^16^. The aim of this study was to assess the feasibility of establishing beagle model of tracheal stenosis induced by cuffed endotracheal intubation. By comparing different cuff pressures and tube sizes in the modeling, the effects of cuff pressure and intubation tube size on the formation of postintubation tracheal stenosis were also elucidated.

## Methods

### Animals

Nineteen adult beagles (10–13 kg, 11–13 month) were purchased from Gaoyao Kangda Laboratory Animal Science and Technology Co., Ltd. This study was approved by the Ethical Committee of the First Affiliated Hospital of Guangzhou Medical University (Ethical Committee 2015 NO. 17), and all methods were performed in accordance with the relevant guidelines and regulations. The animal use protocol had been reviewed and approved by the Animal Ethical and Welfare Committee (Approval NO. IACUC-DB-15-0303). All experiments were performed in accordance with the guidelines and regulations of the Animal Ethical and Welfare Committee.

### Establishing animal model of tracheal stenosis

**Figure 1:**
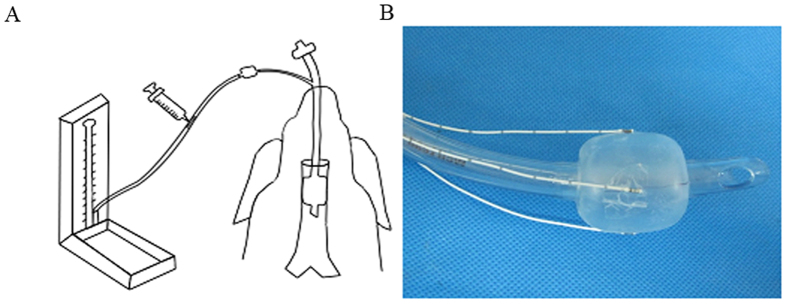
Devices used in the current study. (**A**) Schematic diagram of the device used to create the tracheal stenosis model. The device consists of a three-way tube connected to the tube, a standard medical mercury sphygmomanometer, and a syringe, respectively. Syringe was used to inflate the cuff by injecting air, and the intra-cuff pressure was monitored by the sphygmomanometer. (**B**) Four Codman MicroSensors were attached to the cuff, which was used to directly measure the tracheal wall pressure.

Ketamine (1 mg/kg) was given by intramuscular injection in conjunction with 3% pentobarbital sodium (3%; 0.5 ml/kg) given intravenously to induce anesthesia. The tracheal inner diameters of the dogs were 1.6–1.8 cm, as measured by flexible bronchoscope (BF-160, Olympus, Japan). Cuffed endotracheal intubation was performed under laryngoscope guidance. Cuffed tubes with an internal diameter of 6.5 mm (I.D. 6.5), I.D. 7.0, I.D. 7.5 and I.D. 8.0 (Parker Medical, Welllead, China) were chosen for intubation. The inflated cuff diameter of the tube given above is 20 mm, 22 mm, 25 mm and 26 mm, respectively. The depth of tube insertion was adjusted such that the top of the balloon was 3–4 cm below the glottis. A homemade device was used to monitor and maintain a stable intra-cuff pressure (Fig. 1). The cuffs were inflated to different cuff pressures (100 mmHg, 150 mmHg, or 200 mmHg, respectively) by injecting air. During the 24-hr intubation, 3% sodium pentobarbital was given by intraperitoneal injection at a rate of 0.5 ml/h to maintain anesthesia. Details of the intubation tube size and cuff pressure are shown in Table 1.

### Determine the tracheal wall pressure

Eight animals with the same tracheal inner diameter (1.7 cm) were used for determine the tracheal wall pressure. After the animal was anesthetized and fixed, 4 sizes of cuffed tubes (I.D. 6.5, I.D. 7.0, I.D. 7.5, I.D. 8.0, Hi-Lo, Wellead) and 2 animals for each sized tube were used for intubation. The inflated cuff diameters were 20 mm, 22 mm, 25 mm and 26 mm, respectively. The tracheal wall pressure was measured as described previously^19^. Briefly, four Codman MicroSensors (Johnson & Johnson Medical, UK) were attached to the cuff (Fig. 1B) using 3 M Tegaderm (3 M Health Care, Minnesota, USA). After intubation, the cuff was inflated to different intra-cuff pressures (30~200 mmHg). The pressure (mmHg) measured by the 4 pressure sensors on trachea wall was recorded. Triplicate measurements were performed at each intra-cuff pressure for each animal, and the data was presented as the mean value of six measurements from two animals (mean ± SD). When the four pressure values were similar, and the average on the left and right side wall pressures were recorded as the tracheal wall pressure. The tracheal wall pressures were compared among groups at the same intra-cuff pressure by one-way analysis of variance (ANOVA) with post-hoc Tukey honestly significant difference (HSD) Test. The correlation between intra-cuff pressure and tracheal wall pressure was evaluated by Pearson correlation analysis. P ≤ 0.05 was considered significant.

### Observation and examination

The tracheal lumen was examined by bronchoscope immediately after extubation. The dogs without obvious complications were awake normally. During the observation period after extubation, the occurrence of wheezing, cyanosis and dyspnea was documented.

Airway sections compressed by intubation were examined on a weekly basis by bronchoscopy. The degree of tracheal stenosis was determined by reduction of the tracheal lumen cross-section area: (S − s)/S × 100%, where “s” is the tracheal cross-sectional area after operation, “S” is the area before intubation. Shortness of breath, dyspnea, and a reduction of at least 50% of the visible airway cross-sectional area after extubation indicated a successful model. Dogs exhibiting signs of pain, costal retraction, or significant tracheal stenosis were euthanized by femoral vein injection of pentobarbital sodium (3%; 2.5 ml/kg). Dogs without any signs of breathing difficult or fatal tracheal stenosis were euthanized 21 days after extubation. After euthanasia, tracheal rings compressed by intubation were removed for conventional hematoxylin and eosin staining. Degrees of inflammatory cell infiltration, vascular proliferation, and tracheal cartilage damage were observed and documented using a light microscope (DM4000, Leica, Germany). Degrees of Inflammation were evaluated with the inflammatory score as described previously^14^.

## Results

### Establish the canine model of tracheal stenosis

To obtain the optimal condition to establish the animal model of tracheal stenosis, 6 different combinations (4 sized tubes [I.D. 6.5, I.D. 7.0, I.D. 7.5, I.D. 8.0] and 3 cuff pressures [100 mmHg, 150 mmHg, 200 mmHg]) were tested. The intubation time was set to 24 hr. The detailed conditions for model establishment were summarized in Table 1.

All the animals had stable vital signs during intubation, and were awake from anesthesia at 20–30 min postextubation. No intubation-related complications such as tracheomalacia or tracheal rupture were observed immediately after extubation. However, varying degrees of salivation, reduced mobility and appetite were observed among all animals, especially at 3 days to 1 week after extubation. Four animals intubated with I.D. 8.0 tube and cuff pressure of 200 mmHg (I.D.8.0+P200 group) and 2 out 3 animals in I.D.7.5+P200 group had significant weight loss, mouth breathing, cyanosis, and costal retraction at rest 2 to 3 weeks after extubation, and were euthanatized as appropriate. The remaining dogs had no shortness of breath, wheezing, or breathing difficulties, and were euthanized at 21 days after extubation (Table 1).

### Correlation between intra-cuff pressure and tracheal wall pressure

We attempted to directly measure the tracheal wall pressure (exerted by the cuff) in different sizes of tubes and intra-cuff pressure. As shown in Table 2, there was a highly positive correlation between intra-cuff pressure and tracheal wall pressure among all the 4 sizes of cuffed tubes (the correlation coefficients (r) ranging from 0.9858 to 0.9998, all *P* < 0.001). With the increase of intra-cuff pressure, the tracheal wall pressure gradually increased. However, the ratio of intra-cuff pressure to tracheal wall pressure ranged from 0.44 to 0.9 (at inner-cuff pressure of 200 mmHg) among groups, indicating that the pressure transmit efficiencies (from intra-cuff pressure to endotracheal wall) were different among groups. At the intra-cuff pressure of 70 mmHg to 200 mmHg, the tracheal wall pressures were all significantly lower in I.D. 6.5 group than in other 3 groups (all *p* < 0.01). Notably, at 200 mmHg, the tracheal wall pressures of I.D.7.5 and I.D.8.0 groups were significantly higher than that of I.D.7.0 (both *p* < 0.01). These data suggested a larger sized tube caused a higher tracheal wall pressure.

### Bronchoscopic evaluation

**Figure 2:**
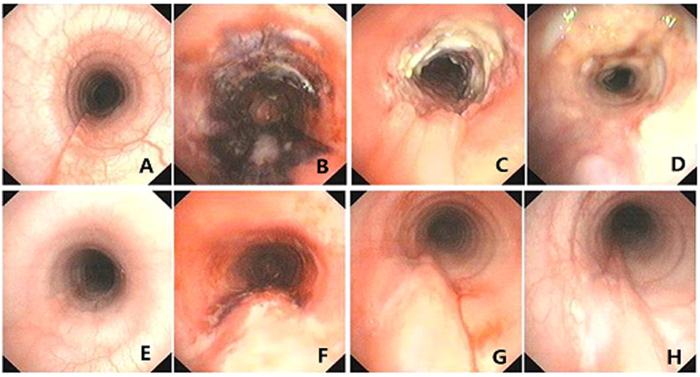
Representative bronchoscopic images. (**A**–**D**) Images of animal intubated with I.D. 8.0 mm tube and cuff pressure of 200 mmHg (I.D.8.0+P200). (**A**) Normal tracheal lumen before intubation. (**B**) Tracheal mucosal ischemic necrosis were observed immediately after extubation. (**C**) Edema, necrotic tissue and granulation tissue were observed at 1 week after extubation. (**D**) Formation of hyperplasic granulation tissue and tracheal stenosis at 2 weeks after extubation. (**E**–**H**) Images of I.D.7.0+P200 animal. (**E**) Normal tracheal lumen before intubation. (**F**) Congestion and mild necrosis were observed immediately after extubation. (**G**) Partial posterior tracheal wall thickened at 1 week after extubation. (**H**) Tracheal mucosa repaired without airway collapse or stenosis at 2 weeks after extubation.

The status of tracheal wall was evaluated by bronchoscope. Representative bronchoscopic images were shown in Fig. 2. In all dogs, edema, congestion, and mucosal necrosis were observed in the tracheal wall immediately after extubation. Fracture or perforation of the trachea ring, and tracheal cartilage exposure did not occur (Fig. 2B and F).

As shown in Table 1, among all the 19 animals, tracheal stenosis were successfully induced in all the 4 animals in I.D.8.0+P200 group (n = 4, modeling rate = 100%) and 2 out 3 animals in I.D.7.5+P200 group (modeling rate = 66.67%) after extubation. One week after extubation, the modeled animals had edema in the tracheal mucosa and increased purulent secretions in the tracheal wall with necrotic tissues. The necrotic mucosal granulation tissue underwent repair and proliferation, and a moderate to server tracheal stenosis (41–56%, Fig. 2C, Table 1) developed. At two weeks after extubation, hyperplasic granulation tissue protruded into the tracheal wall of the modeled animals. The tracheal wall collapsed, and severe tracheal stenosis of 78–91% could be observed (Fig. 2D).

While in the remaining animals, at one week after extubation, it was showed mild thickening of the posterior tracheal wall, and regenerated airway mucosa, but no hyperplasia of granulation tissue or tracheal stenosis (Fig. 2G). At 2 weeks, the edema partially subsided and the airway mucosa repaired without airway collapse or stenosis (Fig. 2H).

### Gross examination

**Figure 3:**
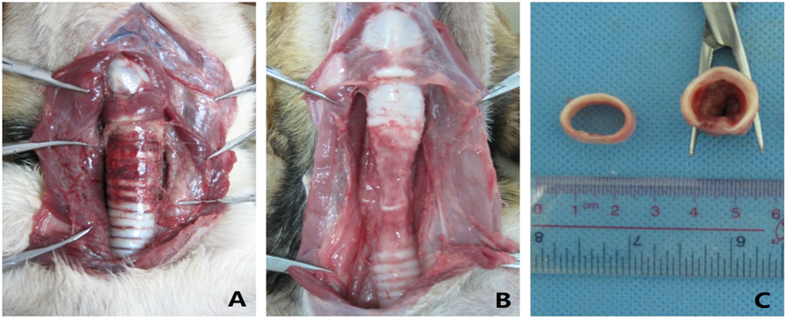
Gross examination of compressed tracheal segment in modeled animal (I.D.8.0+P200). (**A**) Congestion of the external tracheal wall compressed by cuff was observed immediately after extubation. (**B**) Tracheal cartilage underwent necrosis and collapsed at 2 weeks after extubation. (**C**) Hyperplasic granulation tissue led to tracheal occlusion at 2 weeks after extubation.

Gross examination of modeled animal showed that obvious congestion was found immediately after extubation in the segment of the tracheal wall compressed by the cuff. The tracheal cartilage was intact structurally, with no complications such as perforation or tracheomalacia (Fig. 3A).

The mean length of tracheal stenosis in the modeled animals was 1.86 ± 0.40 cm. The tracheal cartilage compressed by the cuff was fractured, accompanied with damage to the supporting structure and collapsed airway wall (Fig. 3B), all of which contributed to severe stenosis and caused fracture of adjacent cartilage rings. While in the remaining dogs, congestion was minimal in the external tracheal wall, and the cartilage was intact without damage or collapse (data not shown).

### Histological assessment

**Figure 4:**
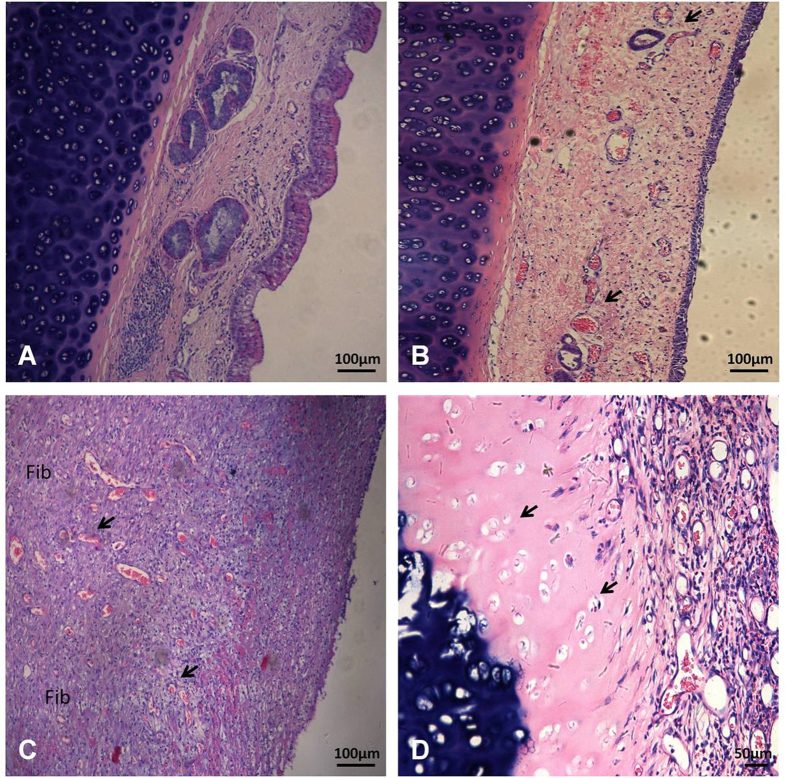
Representative images of histological assessment. (**A**) Normal epithelium and cartilage (100×). (**B**) I.D.7.0+P200 animal, at 3 weeks after extubation. Edema, new capillaries and congestion (black arrow), and inflammatory cell infiltration of the submucosa without granulation tissue growth were observed (100×). (**C**,**D**) I.D.8.0+P200 animal, at 2 weeks after extubation. (**C**) Mucosal epithelium was lost, and a large number of new capillaries (black arrow) and fibroblasts (Fib) were present (100×). (**D**) The perichondrium and chondroblasts were lost, nuclei of cartilages were degenerated on the luminal side (black arrow), hyperplasic granulation tissue and collagen fibers invaded the necrotic cartilage (400×).

Representative histological image of normal epithelium and cartilage was shown in Fig. 4A. In the non-modeled dogs, at 3 weeks after extubation, shedding of the tracheal mucosal epithelium and partial epithelialization, edema, congestion, and inflammatory cell infiltration of the submucosa was noted. These dogs had no granulation tissue growth, and both the tracheal cartilage and the perichondrium were intact (Fig. 4B). The modeled animals had thickened airway walls, while the mucosal epitheliums of the compressed trachea were lost at two weeks after extubation. Parts of the epithelium displayed squamous metaplasia, exudation of submucosal inflammatory cells, excessive hyperplasia of granulation tissue and collagen fibers. In addition, a large number of new capillaries and fibroblasts were observed (Fig. 4C). The perichondrium and chondroblasts were lost, and the cartilage matrix underwent histological acidophilic changes and necrosis. Furthermore, hyperplasic granulation tissue and collagen fibers invaded into the necrotic cartilage tissue (Fig. 4D). In addition, the mean inflammatory score was significantly higher in the modeled animals than in the non-modeled dogs (11.83 ± 0.17 vs. 7.0 ± 0.39, t-test, P < 0.001, Table 1).

## Discussion

In this study, we successfully established a canine model of tracheal stenosis induced by cuffed endotracheal intubation using an I.D. 8.0 mm tube and an intra-cuff pressure of 200 mmHg for 24 hr. The intubation caused congestion, inflammation, and ulceration of the tracheal wall compressed by the cuff immediately after extubation. One week after extubation, significant inflammation and edema of the tracheal mucosa, as well as necrotic tissue and granulation tissue were observed. At two weeks after extubation, excessive hyperplasia of granulation tissue, destruction of the tracheal cartilage, and collapse of the tracheal wall contributed to tracheal stenosis (degree of 78–91%). The histological analysis indicated that excessive hyperplasia of granulation tissue and formation of fibrous tissue resulted in thickening of the tracheal wall, accompanied with ischemic necrosis of the perichondrium and cartilage and damage to the tracheal wall framework. All these data was consistent with the clinical histological observations^20^,^21^. However, the timing of symptoms onset in our model was different from other published tracheal stenosis animal models^22^,^23^. In our model intubation-induced mechanical compression did not damage the tracheal cartilage directly, but induced ischemic injury to the perichondrium and cartilage tissue, leading to necrosis or loss of tracheal cartilage within the following 2 weeks. The time course of pathogenesis is more close to that of the clinical patients with postintubation tracheal stenosis. To our best knowledge, this is the first animal model of tracheal stenosis induced by over-size cuffed endotracheal tubes with high pressure, which is similar to the occurrence of postintubation tracheal stenosis in most of adult critical care patients^24^.

Postintubation tracheal stenosis occurs most frequently in the tracheal region contacted with cuff. It is recommended that the intra-cuff pressure should not exceed 22 mmHg to prevent obstruction of tracheal wall blood flow and ischemic injury^25^. When the cuff pressure is greater than the capillary pressure during intubation, submucosal blood flow is obstructed, leading to ischemic injury to the airway wall^26^. Julian *et al*. showed that when the cuff pressure is maintained at 100 mmHg for 4 hr, the inflammation and injury penetrate down to tracheal cartilage^27^. Histopathological analysis suggested that damage and destruction of the tracheal perichondrium and cartilage are key factors for tracheal stenosis and indicators of poor prognosis^28^,^29^. Tracheal cartilages receive blood supply from the capillary bed on their internal surface. Hence, chondrocyte and chondroblasts are relatively sensitive to oxygen and nutrient deprivation, making tracheal cartilage vulnerable to ischemic injury caused by cuff compression. Ischemic injury results in the destruction of cartilage framework, fibrogenesis and acidophil staining^30^. It has been shown that injuries to the tracheal mucosa and submucosa only result in normal wound healing but not tracheal stenosis^11^, while damages to the tracheal cartilage and connective tissue induce proliferation rather than regeneration, thereby leading to overgrowth of granulation tissue and eventual tracheal stenosis^15^,^28^. The depth of airway wall injury has been shown to be closely correlated with tracheal stenosis^31^.

The risk factors for postintubation tracheal stenosis may involve increasing cuff pressure, over-sized intubation tube, and/or prolonged intubation period^5^,^32^,^33^. The current guideline on endotracheal intubation^1^ indicates that suitable intubation tube sizes for adults are I.D. 7.0, I.D. 7.5, and I.D. 8.0, whereas specification for selecting the optimal tube size is unclear. Karmakar *et al*. recommend a tube size of I.D. 7.0–8.0 for men, while an I.D. 6.5–7.0 for women^34^. A multicenter study^35^ showed that 22.7% obese patients intubated with a tracheal tube >7.5 mm developed tracheal stenosis, compared with 3.3% of those with a tube size ≤7.5 mm, indicating the correlation between tube size and postintubation tracheal stenosis. In our study, as the tracheal diameter, cuff pressure, and intubation time were kept constant, the larger-sized tubes (I.D. 7.5 and I.D. 8.0) were more likely to cause ischemic injury, significant inflammation, excessive hyperplasia of granulation tissue, cartilage destruction and subsequent postintubation tracheal stenosis.

As for the cuff pressure, the current endotracheal intubation guideline^1^ lacks detailed instructions on setting the intra-cuff pressure. Study has shown that 31–95% of critical care patients experience tracheal wall ischemic injury after intubation, and that injury is positively associated with cuff pressure^8^,^32^,^33^. Our study showed that when tube size and intubation time were kept constant, a higher cuff pressure caused greater damage to the tracheal wall, along with significant regional ischemic necrosis and inflammation. Moreover, increasing cuff pressure to 200 mmHg caused hyperplasia of granulation tissue and collapse of the cartilage in the cuff-compressed region, whereas the cuff pressure ≤150 mmHg did not result in granulation tissue growth or cartilage destruction. Our observations also support that high intra-cuff pressure is a risk factor for postintubation tracheal stenosis.

The intra-cuff pressure is not directly equal to the tracheal wall pressure exerted by the cuff^5^. The tracheal wall pressure is associated with inflated cuff size and tracheal diameter, the size of contact area between cuff and tracheal wall and intra-cuff pressure. It has also been suggested that the tracheal wall pressure is influenced by the internal elastic recoil of the cuff radius^36^, the wall thickness, elasticity, and extension of the cuff^37^. Although the tracheal wall pressure is crucial for development of postintubation tracheal stenosis, however, it is difficult to be directly measured clinically. In this study, we directly measured tracheal wall pressure using pressure microsensors on the cuff contacted with the tracheal wall in our canine model. The results showed that intra-cuff pressure was positively correlated with the tracheal wall pressure. In addition, when the intra-cuff pressures were kept constant, the larger size of tube (inflated cuff diameter) was used, the higher the tracheal wall pressure would be. At 200 mmHg, the tracheal wall pressures of I.D.7.5 and I.D.8.0 groups were significantly higher than those of I.D. 6.5 and I.D. 7.0 group. This result explained the observation that at the intra-cuff pressure of 200 mmHg, animals intubated with large-sized tubes (I.D. 7.5 and I.D. 8.0) but not small-sized tubes (I.D. 6.5 and I.D. 7.0) can be successfully established the model of tracheal stenosis. In addition, the modeling rate of I.D. 8.0+P200 group was higher than that of I.D. 7.5+P200 group (100% vs. 66.7%). Therefore, to ensure the success of modeling, we suggested using the condition of I.D.8.0 + 200 mmHg. These results also indicated that over-sized intubation tube is a risk factor for postintubation tracheal stenosis.

There are still some limitations in the current study. First, the sample size is relatively small, and time point of histological images was inconsistent between modeled and non-modeled dogs. We originally set three weeks as the study endpoint to sacrifice animals, however, the modeled animals all died around 2 weeks after extubation (ranging 14–19 days) due to dyspnea or euthanasia. Nevertheless, the results should be comparable between two and three weeks since that all animals would be in the phases of inflammation and collagen fiber proliferation within 2–3 weeks after extubation. Second, the relationship between the contact area, the tracheal wall pressure, and the incidence of postintubation tracheal stenosis is worthy of further investigation. Finally, it should be pointed out that the modeling cuff pressure of 200 mmHg in this study is much higher than that used in clinical endotracheal intubation (generally ≤30 mmHg). We used an extremely high cuff pressure in order to establish the model of postintubation tracheal stenosis with a high successful rate in a short time. The extremely high cuff pressure used in this study may contribute to the phenomenon that the timing for occurrence of tracheal stenosis is more rapid in our model (2–3 weeks after extubation) than in clinical cases (3–6 weeks^38^). However, there were still several similarities between our model and clinical cases. Firstly, there was no intubation-induced rupture or collapse in the tracheal wall immediately after extubation in our model. Instead, the intubation-induced ischemic injury gradually caused the development of hyperplasia of granulation tissue and cartilage structure damage in the tracheal wall, which are similar to those of clinical cases. In addition, in pathophysiology, the proliferations of fibroblasts and capillary endothelial cells as well as the formation of collagen fibers typically peaked at 14 to 21 days after mucosal inflammation injuries, the timing and pathophysiological feathers of which are consistent with those in our histological assessments. Moreover, the histopathological changes of our animal models are in line with the pathological features of human tracheal cartilage with ischemic injury^39^.

In summary, we used endotracheal intubation to establish an animal model of tracheal stenosis. The modeling method possesses a low mortality of animals, a high success rate and reproducibility, which is appropriate to be used in pre-clinical studies. In addition, the histopathological feathers of our model are similar to those of clinical cases. Excessive cuff pressure and over-sized tube are the risk factors for postintubation tracheal stenosis. This model may facilitate further studies on the pathogenesis of postintubation tracheal stenosis, and the development of new techniques for preventing and treating postintubation tracheal stenosis.

## Additional Information

**How to cite this article**: Su, Z. *et al*. A canine model of tracheal stenosis induced by cuffed endotracheal intubation. *Sci. Rep.* **7**, 45357; doi: 10.1038/srep45357 (2017).

**Publisher's note:** Springer Nature remains neutral with regard to jurisdictional claims in published maps and institutional affiliations.

## Figures and Tables

**Table 1:** Outcomes of tracheal intubation and degrees of inflammation.

Beagle No.	Cuff pressure (mmHg)	Tube size (mm)	Tracheal inner diameter (cm)			Length of stenotic area (cm)	Survival time (day)	Inflammatory score*					
				Stenosis (%)				Capillaries			Inflammatory cells		Total scores
								Day 7	Day 14	Dilatation^†^	Number^‡^	Congestion^§^		Invasion**	Number^††^
1	200	8	1.7	47	91	1.4	15	2	2	2	3	3	12
2	200	8	1.6	46	81	2.5	12	2	2	1	3	3	11
3	200	8	1.6	41	78	1.5	21	2	2	2	3	3	12
4	200	8	1.7	56	88	1.8	17	2	2	2	3	3	12
5	200	7.5	1.8	50	83	2.1	14	2	2	2	3	3	12
6	200	7.5	1.7	53	82	1.9	19	2	2	2	3	3	12
7	200	7.5	1.7	—	—	—	21	2	1	2	2	3	10
8	200	7	1.7	—	—	—	21	2	1	2	1	1	7
9	200	7	1.6	—	—	—	21	1	1	2	1	1	6
10	200	7	1.6	—	—	—	21	2	1	2	1	2	8
11	200	6.5	1.6	—	—	—	21	1	1	2	1	2	7
12	200	6.5	1.7	—	—	—	21	1	1	1	2	1	6
13	200	6.5	1.6	—	—	—	21	1	1	1	1	2	6
14	150	8	1.7	—	—	—	21	2	1	1	2	2	8
15	150	8	1.6	—	—	—	21	2	1	2	2	2	9
16	150	8	1.7	—	—	—	21	2	1	1	1	2	7
17	100	8	1.8	—	—	—	21	1	1	2	1	1	6
18	100	8	1.7	—	—	—	21	1	1	1	1	1	5
19	100	8	1.6	—	—	—	21	1	1	1	1	2	6

*Scoring Criteria^14^.

^†^1-mild; 2-marked.

^‡^1-total number of blood vessels seen in five arbitrary views at ×200 being less than 150; 2 -total number of blood vessels 150 or more.

^§^1-mild; 2-marked.

**1-infiltrating inflammatory cells in localized tissue; 2 -infiltrating inflammatory cells in partial tissue; 3 – infiltrating inflammatory cells in entire tissue.

^††^1, 2 and 3-average number of inflammatory cells in five arbitrary views ×400 being less than 50; between 50 and 100, and more than 100, respectively.

**Table 2:** Correlation analysis between intra-cuff pressure and tracheal wall pressure.

Intra-cuff pressure (mmHg)	^∆^Tracheal wall pressure (mmHg)				*P_*1*_
	I.D. 6.5	I.D. 7.0	I.D.7.5	I.D.8.0	
200	87.33 ± 4.04	147.33 ± 13.61^a^	177.00 ± 3.61^a,b^	180.00 ± 2.00^a,b^	<0.001
170	67.33 ± 4.16	125.67 ± 19.66^a^	146.33 ± 6.66^a^	149.67 ± 5.69^a^	<0.001
150	54.33 ± 4.51	109.67 ± 17.04^a^	128.00 ± 6.24^a^	131.00 ± 5.29^a^	<0.001
120	39.00 ± 8.72	83.67 ± 14.47^a^	101.67 ± 4.04^a^	103.00 ± 5.29^a^	<0.001
100	27.00 ± 1.73	68.00 ± 11.53^a^	82.67 ± 2.31^a^	84.33 ± 6.51^a^	<0.001
70	16.33 ± 1.53	43.00 ± 7.21^a^	56.67 ± 5.03^a^	54.67 ± 8.50^a^	<0.001
50	11.33 ± 2.08	30.00 ± 5.29^a^*	39.00 ± 6.08^a^	35.33 ± 6.66^a^	0.001
30	7.00 ± 1.73	13.33 ± 4.04	18.00 ± 2.65	19.33 ± 7.37^a^*	0.0371
Outer diameter of inflated cuff (mm)	20	22	25	26	
^†^Correlation coefficient (r)	0.9858	0.9997	0.9997	0.9998	
^*#*^*P*_*2*_	<0.001	<0.001	<0.001	<0.001	

^∆^2 animals for each group, triplicate measurements at each intra-cuff pressure for each animal. The data was presented as the mean value of six measurements from two animals (mean ± SD).

*P value for intergroup comparison at the same intra-cuff pressure (one-way ANOVA).

^a^*P* < 0.01 *vs*. I.D. 6.5 at the same intra-cuff pressure, ^a^*P < 0.05 *vs*. I.D. 6.5 at the same intra-cuff pressure, ^b^*P* < 0.01 *vs*. compared with I.D. 7.0 at the same intra-cuff pressure, ^c^*P* < 0.01 *vs*. compared with I.D. 7.5 at the same intra-cuff pressure (post-hoc Tukey HSD Test.

^†^Pearson’s correlation coefficient between intra-cuff pressure and tracheal wall pressure.

^#^P value for the Pearson correlation analysis between intra-cuff pressure and tracheal wall pressure.
